# Intraductal papillary mucinous carcinoma of the pancreas associated with pancreas divisum: a case report and review of the literature

**DOI:** 10.1186/s12876-015-0313-3

**Published:** 2015-07-08

**Authors:** Takeshi Nishi, Yasunari Kawabata, Noriyoshi Ishikawa, Asuka Araki, Seiji Yano, Riruke Maruyama, Yoshitsugu Tajima

**Affiliations:** 1 Deparment of Surgery, Matsue Red Cross Hospital, 200 Horo-machi, Matsue, Shimane 690-8506 Japan; 2 Department of Digestive and General Surgery, Shimane University Faculty of Medicine, 89-1 Enyacho, Izumo, Shimane 693-8501 Japan; 3 Department of Organ Pathology, Shimane University Faculty of Medicine, 89-1 Enyacho, Izumo, Shimane 693-8501 Japan

**Keywords:** Intraductal papillary mucinous carcinoma, Pancreas, Pancreas divisum, Ventral pancreas

## Abstract

**Background:**

Pancreas divisum, the most common congenital anomaly of the pancreas, is caused by failure of the fusion of the ventral and dorsal pancreatic duct systems during embryological development. Although various pancreatic tumors can occur in patients with pancreas divisum, intraductal papillary mucinous neoplasm is rare.

**Case presentation:**

A 77-year-old woman was referred to our hospital because she was incidentally found to have a cystic tumor in her pancreas at a regular health checkup. Contrast-enhanced abdominal computed tomography images demonstrated a cystic tumor in the head of the pancreas measuring 40 mm in diameter with slightly enhancing mural nodules within the cyst. Endoscopic retrograde pancreatography via the major duodenal papilla revealed a cystic tumor and a slightly dilated main pancreatic duct with an abrupt interruption at the head of the pancreas. The orifice of the major duodenal papilla was remarkably dilated and filled with an abundant extrusion of mucin, and the diagnosis based on pancreatic juice cytology was “highly suspicious for adenocarcinoma”. Magnetic resonance cholangiopancreatography depicted a normal, non-dilated dorsal pancreatic duct throughout the pancreas. The patient underwent a pylorus-preserving pancreaticoduodenectomy under the diagnosis of intraductal papillary mucinous neoplasm with suspicion of malignancy arising in the ventral part of the pancreas divisum. A pancreatography via the major and minor duodenal papillae on the surgical specimen revealed that the ventral and dorsal pancreatic ducts were not connected, and the tumor originated in the ventral duct, i.e., the Wirsung’s duct. Microscopically, the tumor was diagnosed as intraductal papillary mucinous carcinoma with microinvasion. In addition, marked fibrosis with acinar cell depletion was evident in the ventral pancreas, whereas no fibrotic change was noted in the dorsal pancreas.

**Conclusion:**

Invasive ductal carcinomas of the pancreas associated with pancreas divisum usually arise from the dorsal pancreas, in which the occurrence of pancreatic cancer may link to underlying longstanding chronic pancreatitis in the dorsal pancreas; however, the histopathogenesis of intraductal papillary mucinous neoplasm in this anomaly is a critical issue that warrants further investigation in future.

## Background

Pancreas divisum, the most common congenital anomaly of the pancreas, is the result of non-fusion of the ventral and dorsal pancreatic duct systems. The incidence of pancreas divisum is reported to range from 5 to 10 % in Western countries [1–3], but only from 1 to 2 % in Asia [3–5]. About 20–45 % of patients with pancreas divisum develop clinical symptoms [2, 4], and most of these symptoms are associated with pancreatitis of the dorsal pancreas due to stenosis or a small orifice of the minor duodenal papilla that can block the outflow of pancreatic juice in the dorsal duct into the duodenum [6, 7]. Pancreatic tumors sometimes develop in patients with pancreas divisum, with an incidence of 11.1–12.5 % [8, 9]. The majority of these pancreatic tumors are ductal carcinomas, and intraductal papillary mucinous neoplasm (IPMN) is rare [8, 9]. We herein report a case of intraductal papillary mucinous carcinoma (IPMC) arising in the ventral part of pancreas divisum and review the relevant literature.

## Case presentation

**Fig. 1:**
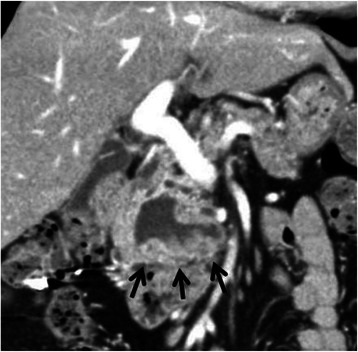
Coronal image of contrast-enhanced computed tomography of the abdomen. A cystic tumor is seen in the head of the pancreas with slightly enhancing mural nodules (arrows)

**Fig. 2:**
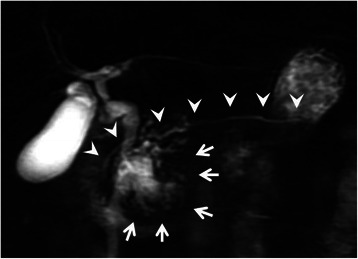
Magnetic resonance cholangiopancreatography. A cystic tumor is seen in the head of the pancreas (arrows). The dorsal pancreatic duct is normal in size and clearly depicted throughout the pancreas (arrow heads)

**Fig. 3:**
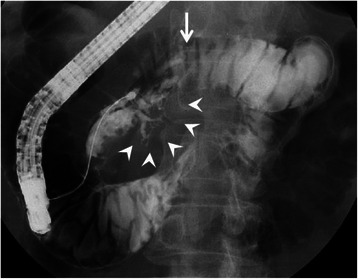
Endoscopic retrograde pancreatography via the major duodenal papilla. A cystic tumor (arrow heads) and a slightly dilated main pancreatic duct, which is abruptly interrupted at the head of the pancreas (arrow), are seen

**Fig. 4:**
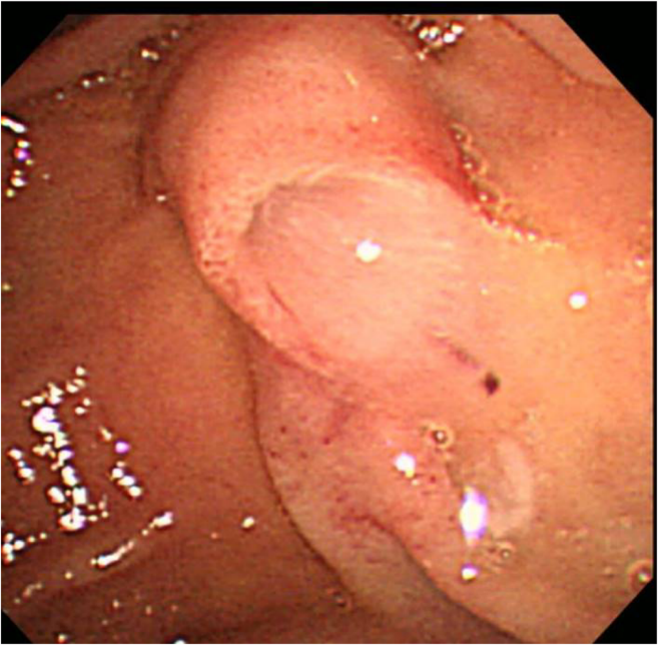
Endoscopic findings of the major duodenal papilla. The major duodenal papilla is enlarged and the orifice is filled with abundant mucin

A 77-year-old woman underwent an annual medical checkup and was diagnosed with a cystic tumor in the pancreas by computed tomography (CT) of the abdomen. The patient was referred to our hospital for further examination. Her medical history included a radical parotidectomy for a parotid gland tumor and a total knee replacement for the right leg. In addition, she was receiving treatment for hypertension and osteoporosis. On admission, she had no clinical symptoms. Her height was 154 cm and her body weight was 61 kg. There was no superficial lymphadenopathy or palpable mass in the abdomen. Her serum amylase level was 211 U/L (normal range; 30–120 U/L), and other biochemical data, including tumor marker levels, fasting plasma glucose, and hemoglobin A1c, were within normal ranges. An upper gastrointestinal endoscopy showed esophageal hiatal hernia and short-segment Barrett’s esophagus. Colonoscopy showed diverticula in the sigmoid colon. Contrast-enhanced abdominal CT scanning demonstrated a cystic tumor in the head of the pancreas measuring 40 mm in diameter with slightly enhancing mural nodules within the cyst (Fig. 1). Magnetic resonance cholangiopancreatography (MRCP) revealed a cystic tumor in the head of the pancreas along with a normal, non-dilated dorsal pancreatic duct throughout the pancreas (Fig. 2). The presence of a connection between the cystic lesion and the main pancreatic duct was unclear. Endoscopic retrograde pancreatography (ERP) via the major duodenal papilla showed a cystic tumor and a slightly dilated main pancreatic duct, but the main pancreatic duct was abruptly interrupted at the head of the pancreas (Fig. 3). The major duodenal papilla was enlarged and the orifice was filled with abundant mucin (Fig. 4). The minor duodenal papilla was normal in size and ERP via the minor papilla was not possible. The diagnosis based on pancreatic juice cytology was “highly suspicious for adenocarcinoma,” suggestive of an intraductal papillary mucinous carcinoma (IPMC) arising in the ventral pancreas of pancreas divisum. The patient underwent a pylorus-preserving pancreaticoduodenectomy (PPPD) with regional lymphadenectomy. The postoperative course was uneventful, except for a Grade A pancreatic fistula (staged according to the International Study Group on Pancreatic Fistula clinical criteria [10]), and the patient was discharged on postoperative day 29.

**Fig. 5:**
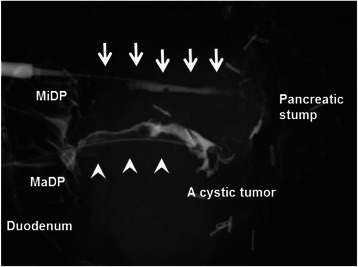
Pancreatography via the major duodenal papilla (MaDP) and minor duodenal papilla (MiDP) on the surgical specimen. There is no connection between the dorsal pancreatic duct (arrows) and the ventral pancreatic duct (arrow heads). A cystic tumor is connected to the ventral pancreatic duct

**Fig. 6:**
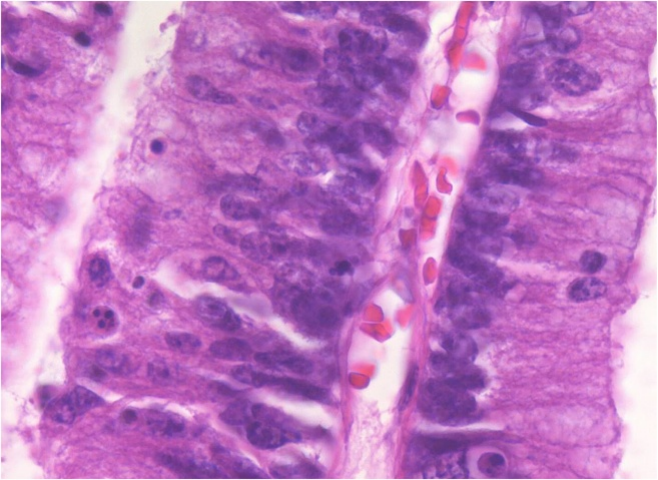
Microscopic findings of the cystic tumor. The tumor is composed of atypical epithelial cells showing nuclear enlargement, clear nucleoli, and disordered polarity (hematoxylin and eosin, magnification 400×)

**Fig. 7:**
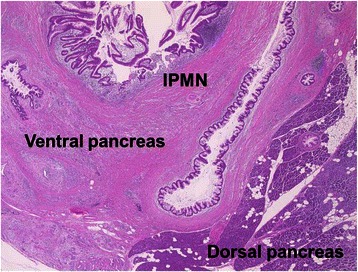
Microscopic findings of the head of the pancreas. The pancreas bearing the tumor shows marked fibrosis, which is clearly distinguished from the neighboring pancreas. The normal pancreas i.e., the dorsal pancreas, is rich in adipose tissue (hematoxylin and eosin, magnification 200×)

A pancreatography via the major and minor duodenal papillae on the surgical specimen revealed no connection between the ventral and dorsal pancreatic duct systems (Fig. 5). Macroscopically, a multilocular cystic tumor, 40 × 35 × 25 mm in size, with abundant accumulation of mucin was identified in the ventral pancreas. Microscopically, the tumor was composed of atypical epithelial cells showing nuclear enlargement, clear nucleoli, and disordered polarity (Fig. 6). They formed prominent papillary structures. The Mib-1 index was up to 80 %. The tumor cells slightly progressed into the main pancreatic duct. Finally, the tumor was determined to be a mixed type IPMC (well-differentiated adenocarcinoma) with partial microinvasion. The pancreas bearing the tumor was drained by the pancreatic duct, which opened into the major papilla, suggesting that it was the ventral pancreas. The region surrounding the IPMC was mainly composed of fibrous tissue, which was clearly distinguished from the normal pancreas (Fig. 7). The normal pancreas was relatively rich in adipose tissue and the islets of Langerhans were typically oval in shape, features consistent with the dorsal pancreas. Furthermore, the pancreatic duct in the region of the normal pancreas was linked to the minor papilla, also suggesting a dorsal pancreas origin.

## Discussion

The pancreas is formed from the dorsal and ventral buds of the foregut. Between the 6th and 8th week of embryological life, the ventral bud rotates behind the duodenum to reach below and behind the head of the dorsal pancreas. The ventral bud forms the remainder of the head and uncinate process of the pancreas, while the dorsal bud develops into the remainder of the head and the whole of the body and tail of the pancreatic gland [6]. The two ductal systems fuse so that the pancreatic gland drains almost entirely through Wirsung’s duct into the major duodenal papilla, and the distal dorsal duct regresses and drains as Santorini’s duct through the minor duodenal papilla [6]. When the dorsal and ventral pancreatic duct systems fail to join during embryogenesis, the condition is called “pancreas divisum,” and it results in inadequate pancreatic drainage because the bulk of the pancreatic gland drains through Santorini’s duct via the minor duodenal papilla [11]. Warshaw et al. [12] have classified pancreas divisum into three types: type 1: the ventral pancreatic duct (Wirsung’s duct) and dorsal pancreatic duct (Santorini’s duct) are completely divided, which is the most common type, accounting for up to 70 % of cases; type 2: an absent ventral pancreatic duct, accounting for up to 20–25 % of cases; and type 3: a filamentous connection between the two duct systems. In our case, pancreas divisum was classified as type 1 based on ERP, MRCP, and pathological findings. However, there was a slight chance that it was an incomplete divisum and the connection between the ventral and dorsal pancreatic duct systems was obscured by the coexisting IPMC.

IPMN associated with pancreas divisum is rare. Recently, Zippi et al. [13] summarized 13 cases of IPMN associated with pancreas divisum. To identify potentially relevant articles related to IPMN associated with pancreas divisum, we performed a literature review by querying PubMed and MEDLINE with the search term “pancreas divisum” and “intraductal papillary mucinous tumor” or “intraductal papillary mucinous neoplasm”. In addition, references from these articles were also assessed for additional relevant materials. As a result, 15 cases of IPMN associated with pancreas divisum, including our case, were identified in the English literature [8, 14–26] (Table 1). Based on these reports, the following clinical characteristics were identified: 1) female predominance (80 %), 2) predominance of type 1 (complete pancreatic divisum) (80 %), 3) tumor location in the dorsal pancreas (80 %) rather than in the ventral pancreas, and 4) a predominance of the branch-duct type (60 %) over the main-duct type. More than half of the patients presented with clinical symptoms, such as back pain or epigastric pain, and two patients’ symptoms were associated with pancreatitis. In general, most cases of pancreas divisum are asymptomatic and some may present with clinical symptoms related to pancreatitis [6]. In patients with IPMN, mucin-producing tumors may cause an accumulation of mucin in the pancreatic ducts and subsequent obstructive pancreatitis. Although our patient had no symptoms, pancreatic IPMNs arising in patients with pancreas divisum may modify the clinical symptoms of the patients, because dorsal pancreatitis associated with relative stenosis of the minor papilla and pancreatitis due to IPMN may interact with each other. In our case, fibrosis of the pancreas associated with acinar cell degeneration was remarkable in the ventral pancreas, but the bulk of the pancreatic gland, i.e., the dorsal pancreas, was normal without any findings of pancreatitis. This may be the reason why our patient showed no clinical symptoms.

**Table 1:** IPMNs of the pancreas associated with pancreas divisum

Authors [ref.]	Year	Patient age (years)	Sex	Type of pancreas divisum^a^	Type of IPMN	Tumor location	Tumor location based on embryology	Tumor histology	Operation	Clinical symptoms
Origuchi [14]	1996	82	F	1	Branch	Head	Dorsal	Adenoma	(Autopsy)	Icterus (due to other disease)
Thayer [15]	2002	71	F	1	MD	Head-tail	Dorsal	Carcinoma	DorP	None
Yarze [16]	2003	33	F	1	Branch	Head	Dorsal	Adenoma	PD	Epigastralgia, nausea
Sakurai [17]	2004	74	M	2	Branch	Head	Dorsal	Adenoma	PPPD	None
Sakate [18]	2004	34	M	1	Branch	Head	Ventral	Adenoma	PPPD	Back pain, epigastralgia
Kamisawa [8]	2005	63	F	1	Branch	Head	Dorsal	Unknown	Unknown	Unknown
Talbot [19]	2005	51	F	1	Branch	Head	Dorsal	Carcinoma	DorP	None
Akizuki [20]^c^	2006	75	F	1	Branch	Body, Tail	Dorsal, Dorsal	Adenoma	CP & PR	Back pain
Scatton [21]	2006	45	M	1	MD	Head-tail	Dorsal	Adenoma	DorP	Episode of pancreatitis
Sterling [22]	2007	70	F	3	MD	Head-tail	Dorsal	Carcinoma^b^	None	Epigastralgia, body weight loss
Santi [23]^c^	2010	74	F	1	Branch	Head, Tail	Dorsal, Ventral	Unknown	None	None
Ringold [24]	2010	65	F	1	MD	Head-tail	Dorsal	Adenoma^b^	None	None
Nakagawa [25]^d^	2013	70	F	2	Branch	Head-tail	Dorsal	Unknown	None	Relapsing acute pancreatitis
Gurram [26]	2014	39	F	1	MD	Head	Ventral	Adenoma	PD	Epigastralgia
Present case		74	F	1	Mixed	Head	Ventral	Carcinoma	PPPD	None

IPMN: intraductal papillary mucinous neoplasm, ^a^type of pancreas divisum according to Warshaw’s classification, ^b^cytological diagnosis, ^c^two tumors existed in the pancreas, ^d^multiple tumors existed in the pancreas, Branch: branch-duct type, MD: main-duct type, Mixed: mixed type, DorP: dorsal pancreatectomy, PD: pancreaticoduodenectomy, PPPD: pylorus-preserving pancreaticoduodenectomy, CP: central pancreatectomy, PR: partial resection

The treatment for IPMN with pancreas divisum depends both on the malignant potential of the IPMN and the symptoms related to pancreas divisum. Our patient showed enhancing mural nodules within the cystic tumor of the pancreas on contrast-enhanced CT examination, and pancreatic juice cytology results were highly suspicious for adenocarcinoma. A surgical resection was thus indicated for the patient according to the international consensus guidelines for IPMN proposed by International Association of Pancreatology [27]. Among reported cases of IPMN with pancreas divisum, operative procedures vary, with some patients undergoing minimally invasive surgery, such as a dorsal pancreatectomy (Table 1). With regard to the surgical management of IPMNs, the extent of pancreatic resection and lymph node dissection has not been standardized because the malignant potential of pancreatic IPMNs varies. Therefore, it is important to plan an adequate operation on an individual patient basis. Although organ-preserving pancreatectomy, such as duodenum-preserving pancreatic head resection, pancreatic head resection with segmental duodenectomy, or ventral pancreatectomy, could be the treatment of choice for benign or low-grade malignant lesions of the pancreas, PPPD was performed in our patient because the cystic tumor of the pancreas was strongly considered to be IPMC based on preoperative investigations and because a curative pancreatectomy provides a favorable prognosis for patients with IPMC [28, 29].

The etiologic relationship between pancreas divisum and IPMN is an extremely interesting issue. Most pancreatic ductal carcinomas arising in patients with pancreas divisum develop in the dorsal pancreas, and longstanding pancreatitis in the dorsal pancreas caused by relative stenosis of the minor duodenal papilla has been considered to be a predisposing factor for ordinal pancreatic cancer [8, 30]. In our patient, IPMC developed in the ventral pancreas. Because the ventral pancreas drains through the major papilla, ventral pancreatitis is very rare in patients with pancreas divisum [31, 32]. Although marked fibrosis with acinar cell degeneration was evident in the ventral pancreas in our patient, it might be due to chronic obstructive pancreatitis related to the accumulation of mucin in the pancreatic ducts. Talamini et al. [33] have suggested that IPMN is the cause of chronic pancreatitis and not vice versa. Although it is difficult to elucidate the etiologic relationship between pancreas divisum and IPMN, the co-existence of these two unique disorders may have no strong relevance, and the histopathogenesis of IPMNs in patients with this anomaly is a critical issue that warrants further investigation in future.

## Conclusion

Longstanding obstructive pancreatitis in the dorsal pancreas due to obstruction of pancreatic juice outflow into the duodenum through the minor duodenal papilla might be a risk factor for ordinal pancreatic cancer arising in the dorsal pancreas in patients with pancreas divisum, but the relationship between pancreas divisum and IPMN is currently unclear. A large case series study would be needed to clarify the etiology of IPMN arising in patients with pancreas divisum.

## Consent

Written informed consent was obtained from the patient for publication of this case report and any accompanying images. A copy of the written consent is available for review by the Editor in Chief of this journal.

## Abbreviations

IPMN: Intraductal papillary mucinous neoplasm
CT: Computed tomography
MRCP: Magnetic resonance cholangiopancreatography
IPMC: Intraductal papillary mucinous carcinoma
ERP: Endoscopic retrograde pancreatography
PPPD: Pylorus-preserving pancreaticoduodenectomy
